# Targeting in vitro vasculogenic mimicry and associated stemness transcriptional signature in human ovarian cancer cell models: new emerging roles of caffeic acid phenethyl ester synthetic analogs

**DOI:** 10.3389/fphar.2026.1787101

**Published:** 2026-03-17

**Authors:** Mohamed Touaibia, Anes Boudah, Alain Zgheib, Bogdan Alexandru Danalache, Borhane Annabi

**Affiliations:** 1 Département de Chimie et de Biochimie, Université de Moncton, Moncton, NB, Canada; 2 Laboratoire d’Oncologie Moléculaire, Département de Chimie, Université du Québec à Montréal, Montreal, QC, Canada

**Keywords:** caffeic acid phenethyl ester, cancer stem cells, chemoresistance, ovarian cancer, vasculogenic mimicry

## Abstract

**Introduction:**

Cancer stem cells (CSC) can sustain tumor growth and therapeutic resistance in part through their contribution to vasculogenic mimicry (VM) in ovarian cancers. Pharmacological targeting of CSC-associated transcriptional programs could represent a promising strategy to overcome recurrence and metastasis. While preclinical studies show caffeic acid phenethyl ester (CAPE), a plant-derived metabolite, can sensitize tumors to chemotherapy and radiotherapy, little is known about its anti-VM properties.

**Methods:**

CAPE (1) and four closely related synthetic analogs were evaluated for their ability to inhibit *in vitro* VM on Matrigel and to remodel CSC molecular signature at the transcriptomic level in human SKOV3 ovarian adenocarcinoma cells and in human ES-2 ovarian clear cell carcinoma. The best analogs were further used for transcriptomic profiling of an 89-gene CSC panel using hierarchical clustering and differential expression analysis.

**Results:**

While CAPE (1) and analogs (2–3) were ineffective, the ketone analog (4) with 3,4-dihydroxy substitution exerted a moderate, pathway-selective effect, reducing VM completely in ES-2 cells, but only partially in SKOV3 cells. In contrast, the ketone analog (5) with 2,5-dihydroxy substitution induced a profound inhibition of VM in both cell models tested and of CSC identity, strongly repressing stemness markers *PROM1*, *ABCG2*, *ALDH1A1*, and pluripotency network regulators *SOX2*, *NANOG*, *LIN28*, alongside broad inhibition of Notch/Wnt/Hedgehog developmental signaling and EMT drivers. Both analogs inhibited serum-induced chemotactic cell migration.

**Conclusion:**

These divergent profiles suggest that CAPE’s synthetic ketone analogs (4) and (5) differentially regulate CSC networks and VM, with (5) producing a coordinated reduction of stemness and plasticity molecular signatures. These findings highlight mechanistic divergence in CSC-targeting strategies that can be attributed solely to the dihydroxy substitution pattern of the two analogs. As effective CSC phenotypic depletion is critical in preventing recurrence and metastasis in ovarian cancer, ketone analog (4) may serve as a selective modulator in contexts requiring partial CSC inhibition. Ketone analog (5) broad pharmacological suppression of CSC programs could position it as a promising candidate for combination therapy to overcome VM-driven chemoresistance.

## 1 Introduction

Caffeic acid phenethyl ester (CAPE) is widely recognized as a plant-derived metabolite, despite its frequent association with bee-derived propolis (Murtaza et al., 2014). Its biosynthetic origin is fundamentally botanical, as CAPE arises from caffeic acid, a hydroxycinnamic acid produced through the phenylpropanoid pathway in plants (Yu et al., 2024). Structurally, CAPE is a polyphenolic phenylpropanoid, a category that encompasses numerous plant secondary metabolites with established biological activities (Pérez et al., 2023). Consistent with this classification, CAPE exhibits antioxidant, anti-inflammatory, and anticancer properties characteristic of plant-derived polyphenols (Natarajan et al., 1996; Wang et al., 2010; Armutcu et al., 2015).

While CAPE displays a broad spectrum of bioactivities, it yet suffers from limitations such as metabolic instability, rapid hydrolysis, and variable bioavailability, the development and study of CAPE analogs has therefore become an important research direction. Structural analogs allow systematic exploration of structure-activity relationships, enabling the identification of chemical modifications that enhance potency, selectivity, pharmacokinetic behavior, or cellular uptake. Moreover, CAPE’s well-defined phenylpropanoid scaffold provides a versatile platform for designing derivatives with improved therapeutic profiles or reduced off-target effects and offers a strong molecular and preclinical rationale for anticancer development (Beauregard et al., 2015; Lin et al., 2015; Chiao et al., 1995; Chung et al., 2013). For these reasons, CAPE analogs serve as valuable tools for probing molecular mechanisms, optimizing drug-like properties, and advancing CAPE-based chemopreventive or therapeutic strategies.

Translationally, CAPE’s pleiotropy reduces the likelihood of single-pathway resistance and creates synthetic vulnerabilities exploitable in combination regimens, but major development challenges remain, chiefly metabolic instability, limited human safety and dosing data, and the need to profile off-target effects (Taysi et al., 2023; Wu et al., 2024). A pragmatic preclinical roadmap therefore emphasizes rigorous mechanism validation, formulation and pharmacokinetic optimization, efficacy testing in orthotopic and patient-derived xenograft models including immune-competent settings, and comprehensive safety/toxicology and drug-drug interaction studies prior to clinical translation. Interestingly, *in vivo* rodent studies show tumor growth delay and enhanced responses when CAPE is combined with chemotherapy or radiation, supporting its potential as an adjuvant (Murtaza et al., 2014). Overall, studying CAPE and optimizing subsequent synthetic analogs could help identify new lead compounds with more robust preclinical anticancer activity, particularly against chemoresistance molecular signatures.

Recently, vasculogenic mimicry (VM) and a cancer stem cells (CSC) molecular signature have been recognized as major contributors to chemoresistance in ovarian cancer (Ayala-Domínguez et al., 2019; Lim et al., 2020; Wilczyński et al., 2022). Therapy-resistant niches and sustained cellular programs that directly counteract cytotoxic treatments have been shown to support tumor-derived, endothelial-like channels that maintain perfusion and nutrient supply when angiogenesis is inhibited or under chemotherapy-induced hypoxia, thereby protecting subpopulations from drug exposure and enabling rapid repopulation after treatment (Yang et al., 2025; Haibe et al., 2020). CSC possess intrinsic resistance mechanisms, express high levels of ABC family drug-efflux transporters, exhibit enhanced DNA-damage response and repair pathways, elevated anti-apoptotic signaling, and a tendency toward quiescence or slow cycling (Safa, 2020; Zhou et al., 2021; El-Tanani et al., 2025). Collectively, these phenotypes are mechanistically linked through hypoxia and epithelial-mesenchymal transition (EMT) programs which promote both VM formation and CSC maintenance, creating a self-reinforcing microenvironment that fosters invasion, metastasis, and therapeutic escape (Haddadin and Sun, 2025).

VM structures and CSC also remodel the extracellular matrix and secrete pro-survival cytokines and proteases among which VEGF, IL-6, and matrix metalloproteinases (MMPs), which impair drug penetration, activate paracrine survival signaling in neighboring cells, and broaden resistance beyond the CSC compartment (Luo et al., 2020; López de Andrés et al., 2020). This biology explains frequent relapse in ovarian cancer and prompts for new therapeutic strategies that simultaneously dismantle the protective niche and eradicate the CSC reservoir through inhibition of stemness pathways. Recently, new peptide-drug conjugates were designed for precise targeting of SORT1-positive ovarian cancer stem-like cells (Demeule et al., 2022), and SORT1-mediated VM in the tumor microenvironment of ovarian ES-2 clear cell carcinoma cells (Charfi et al., 2021), and are currently in phase-1 clinical trial against ovarian cancer (Winer et al., 2025). VM and the CSC molecular signature therefore appear linked since they arise from overlapping transcriptional and signaling programs that confer cellular plasticity, survival, and niche-forming capacity in solid tumors.

Here, we hypothesised that the pharmacological properties of CAPE and four closely related synthetic analogs, encompassing ester, ketone, and bioisosteric modifications can suppress *in vitro* VM phenotype and associated CSC molecular signature, both related to chemoresistance.

## 2 Materials and methods

### 2.1 Reagents and cell lines

Dimethyl sulfoxide (DMSO), Matrigel, and bovine serum albumin were sourced from MilliporeSigma (Oakville, ON, Canada). The human ES-2 ovarian clear cell carcinoma cell line was purchased from the American Type Culture Collection (Manassas, VA, United States). The human SKOV3 ovarian adenocarcinoma cell line was purchased from Cell Biolabs (San Diego, CA, United States). All cell monolayers were cultured according to the vendor’s instructions with McCoy’s 5a Modified Medium for ES-2 cells (Wisent, 317–010-CL), and DMEM medium (Wisent, 319–005-CL) for SKOV3 cells both containing 10% fetal bovine serum (Life Technologies, 12,483–020), 100 U/mL penicillin, and 100 mg/mL streptomycin (Wisent, 450–202-EL) (Sicard et al., 2021). For experiments, cells from frozen aliquots were seeded and subcultured for 5–10 passages. ES-2 is a subtype of epithelial ovarian cancer (EOC) known for its relative resistance to standard platinum-based chemotherapy and poor prognosis. SKOV3 is a human EOC cell line resistant to tumor necrosis factor and to other cytotoxic drugs such as diphtheria toxin, cisplatin, and adriamycin.

### 2.2 *In vitro* vasculogenic mimicry assay

Vasculogenic mimicry (VM) was assessed *in vitro* using Matrigel to monitor 3D capillary-like structures formation. In brief, each well of a 96-well plate was pre-coated with 50 μL of Matrigel. ES-2 or SKOV3 cell suspension in culture media (1.8 × 10^4^ cells/200 μL) was then seeded on top of gelified Matrigel. Tested CAPE plant metabolite and CAPE synthetic analogs were added to the cell culture media and incubated at 37 °C in a CO_2_ incubator. Pictures were taken over time using a digital camera coupled to a phase-contrast inverted microscope. VM parameters were quantified using either the Wimasis Analysis software (Cordoba, Spain) or the ImageJ Software (Uthamacumaran et al., 2021).

### 2.3 Synthesis of CAPE’s analogs

The analog featuring an elongated side chain with an additional methylene unit (analog 2) was synthesized following a previously reported procedure (Sanderson et al., 2013). The ester bioisostere bearing an oxadiazole heterocycle (analog 3) was prepared according to established methods (Robichaud et al., 2025). The 3,4-dihydroxyphenyl ketone (analog 4) was synthesized using the same synthesis strategy as that used for the corresponding 2,5-dihydroxy ketone analog (analog 5) (Giordano et al., 2025). Full experimental details and complete characterization data of analog (4) are provided in the Supporting Information appended.

### 2.4 Total RNA extraction, cDNA synthesis, and real-time quantitative PCR

Total RNA was extracted from ES-2 and SKOV3 cells using TRIzol, following the manufacturer’s recommendations (Life Technologies, Gaithersburg, MD, United States). Total RNA concentration was measured using a NanoPhotometer P330 (Implen) and 2 μg was reverse-transcribed into cDNA using a high-capacity cDNA reverse transcription kit (Applied Biosystems; Foster City, CA, United States). Samples were prepared with primer sets and SsoFast EvaGreen Supermix (Bio-Rad; 1,725,204). The following QuantiTect primer sets were provided by QIAGEN: PROM1 (Hs_PROM1_1_SG, QT00075586), CXCL8 (Hs_CXCL8_1_SG, QT000000322), ABCG2 (Hs_ABCG2_1_SG, QT00073206), SOX2 (Hs_SOX2_1_SG, QT00237601), GAPDH (Hs_GAPDH_2_SG QT01192646) and PPIA (Hs_PPIA_4_SG QT01866137). Gene expression was quantified by RT-qPCR on a CFX Connect (Bio-Rad) with the Bio-Rad CFX manager Software version 3.0. The relative RNA quantities of each target gene were normalized against two housekeeping genes, *GAPDH* and *PPIA*, using the standard 2^−ΔΔCq^ method.

### 2.5 Human transcription factors PCR arrays

Premade RT^2^ Profiler PCR arrays for Human Cancer Stem Cells (PAHS-176Z) were purchased from QIAGEN and were used following the manufacturer’s instructions. Briefly, the genomic DNA was eliminated before reverse-transcribing 0.5 μg of total RNA using the RT^2^ First Strand Kit (QIAGEN, 330,404). Each plate was used to assess one cDNA sample prepared with the RT^2^ SYBR Green qPCR Mastermix (QIAGEN, 330,502). The relative expression analysis of 84 genes as well as controls was done through the GeneGlobe analysis center, a website provided by QIAGEN (https://geneglobe.qiagen.com/us/analyze), using the standard fold change 2^−ΔΔCq^ method. Fold regulation was used in the figures; for upregulated genes, fold regulation is the same as fold change, but, for downregulated genes, fold regulation is calculated as 1/(fold change).

### 2.6 In silico analysis of transcript levels in clinical ovarian cancer tissues

Gene Expression Profiling Interactive Analysis (GEPIA) was used to examine RNA sequencing expression data from ovarian cancer (n = 426) and compared to normal tissue (n = 88), using data sets from The Cancer Genome Atlas (TCGA) and the Genotype-Tissue Expression (GTEx) databases (Tang et al., 2019). Differential gene expression analysis was performed using one-way ANOVA, with disease state as grouping variable for box plot visualization.

### 2.7 Real-time cell migration assay

Cell migration was assessed using the xCELLigence Real-Time Cell Analyzer (RTCA) Dual-Plate (DP) Instrument (Roche Diagnostics, Laval, QC, Canada), following the manufacturer’s protocol. Prior to seeding, the underside of each well in the upper chamber of a CIM-Plate 16 was coated with 0.15% gelatin in PBS and incubated for 1 h at 37 °C. A total of 2.5 × 104 ES-2 cells per well were seeded into the upper chamber and incubated at 37 °C in a humidified atmosphere containing 5% CO2. The lower chamber was filled with either serum-free or serum-enriched medium containing the vehicle (DMSO), analogs (4) or (5). After a 30-min adhesion period, cell migration was monitored in real time for 3 h, with impedance measurements recorded every 30 min. Impedance values, expressed as the Cell Index, reflect the number of cells that migrated through the membrane. All experiments were performed in quadruplicate wells to ensure reproducibility.

### 2.8 Statistical analysis

Data and error bars are expressed as mean ± SD of three independent experiments unless otherwise stated. Statistical analysis was conducted using the non-parametric tests Mann–Whitney U-test (for two-group comparisons) or Kruskal–Wallis test followed by a Dunn Tukey’s post-test (for data with three groups and more). *P* < 0.05 (*) values are considered significant and are indicated in the figures.

## 3 Results

### 3.1 Structural rationale for the CAPE-based synthetic analogs series (1–5)

**FIGURE 1:**
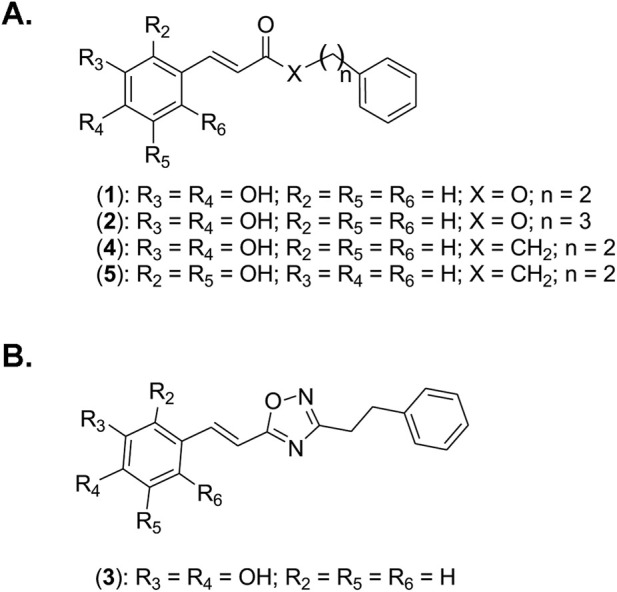
Chemical structures of Caffeic Acid Phenethyl Ester (CAPE) and directly related synthetic analogs 2-5 evaluated in this study. Selected derivatives were generated and used to explore structure-activity relationships. **(A)** Ester- and ketone-based CAPE (1) analogs with modifications in linker nature (X = O or CH_2_), spacer length (n), and hydroxyls substitution pattern on the aromatic ring. **(B)** Bioisosteric analog of CAPE (1) in which the ester function is replaced bye an oxadiazle heterocycle.

The choice of the four synthetic analogs evaluated in this study is based on a rational structure-activity relationship approach centered on CAPE (1) (Figure 1A). CAPE (1) combines a catechol motif (hydroxyl groups in positions 3 and 4) with an ester function linking caffeic acid to a phenylethyl chain. Synthetic ester analog 2 (Figure 1A) is a CAPE (1) analog with an additional methylene group in the side chain. This modification aims to evaluate the influence of chain length and flexibility on biological activity. Synthetic analog 3 (Figure 1B) is an oxadizole bioisostere of CAPE (1) in which the ester function is replaced by an oxadiazole heterocycle, to investigate the role of this function while potentially improving metabolic stability. Synthetic ketone analogs 4 and 5 (Figure 1A), in which the oxygen of the ester was replaced by a methylene group, retained the original 3,4-dihydroxylated substitution of CAPE (1) for 4, while hydroxyls at positions 2,5 for 5 allowed the impact of the topology of the phenolic ring on their activity.

### 3.2 In silico physicochemical profiling of the CAPE-based synthetic analogs

Key physicochemical descriptors were computed for CAPE (1) and its closely related synthetic analogs (2–5) (Table 1) (Daina et al., 2017). All had moderate molecular weights (MW: 282–308 g/mol) and met the main criteria for drug-likeness, in particular Lipinski’s rule, suggesting compatibility with measurable cellular biological activity (Table 1) (Lipinski et al., 2001). CAPE (1) had a topological polar surface area (TPSA) of 66.76 Å^2^ and a LogP of 3.09, which reflects a favourable balance between polarity and lipophilicity, which generally are associated with good cell permeability (Veber et al., 2002). Analog (2), which differs from CAPE (1) in the lengthening of the alkyl chain, showed a moderate increase in LogP (3.43) and the number of rotatable bonds (*n*-ROTB), with no change in TPSA. This increase in molecular flexibility may possibly influence interaction with biological membranes and intracellular distribution. In contrast, the bioisostere (3) is characterized by a higher TPSA (79.38 Å2) and an increased number of hydrogen bond acceptors (n-HBA), reflecting enhanced polarity. Such characteristics are typically associated with improved aqueous solubility, but may limit passive diffusion across cell membranes, which is therefore likely to impact intracellular activity (Pajouhesh and Lenz, 2005). The ketone analogs (4) and (5) possess a particularly balanced physicochemical profile as reflected by their lower TPSA (57.53 Å2) and a moderate LogP (∼3.3). This suggests a more facilitated cell penetration property while maintaining sufficient polar interaction capacity. These parameters are considered favorable for biological activities involving complex intracellular mechanisms, such as the regulation of tumor stemness and VM that were next assessed (Waring, 2009; Lovering et al., 2009).

**TABLE 1:** In silico physicochemical profiling of CAPE (1) and of the CAPE-based synthetic analogs series (2–5)*.*

Molecules	MW (g/Mol)	*n*-ROTB	*n*-HBA	*n*-HBD	LogP	TPSA (Å^2^)
CAPE (1)	284.31	6	4	2	3.09	66.76
(2)	298.33	7	4	2	3.43	66.76
(3)	308.33	5	5	2	3.09	79.38
(4)	282.33	6	3	2	3.37	57.53
(5)	282.33	6	3	2	3.30	57.53

### 3.3 3,4- and 2,5-dihydroxy ketone analogs (4) and (5) differentially disrupt vasculogenic mimicry networks in ES-2 and SKOV3 ovarian cancer cells

**FIGURE 2:**
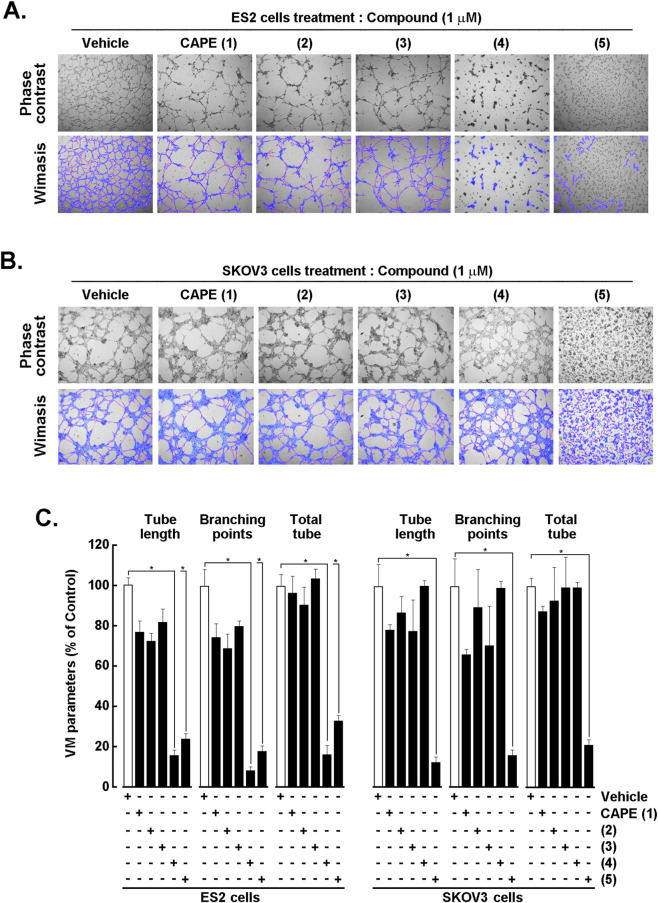
Effects of CAPE (1) and its analogs (2–5) on in vitro vasculogenic mimicry in ovarian cancer cell models. Representative images and Wimasis-processed overlays of the impact of the vehicle, CAPE (1), and analogs (2–5) (1 μM) on capillary-like structure formation in **(A)** ES-2 and **(B)** SKOV3 ovarian cancer cells. **(C)** VM parameters for tube length, branching points, and total tubes for both ES-2 and SKOV3 cells under each treatment. VM parameters expressed as percent of control. Data are the mean ± SD of 3 independent experiments, and asterisks (*) indicate statistically significant differences *versus* the vehicle (DMSO) with *P* < 0.05.

Tumor-cell VM, the formation of perfusable, capillary-like networks by malignant ovarian cancer cells, is believed to support nutrient delivery and metastatic spread (Fouladzadeh et al., 2021). Because VM relies on adhesion, cytoskeletal dynamics, and pro-inflammatory signaling (Dang et al., 2024), we tested whether CAPE (1) and closely related synthetic analogs (2–5), attenuated VM architecture across two distinct ovarian cancer backgrounds. Representative phase contrast images (Figures 2A,B, upper panels) alongside Wimasis segmentation analysis of VM networks (Figures 2A,B, lower panels) after treatment with the vehicle, CAPE (1), or analogs (2–5) are shown. In both cell lines, CAPE (1), (2) and (3) had no measurable effects on tube length, branching points, and total tubes network complexity compared with the vehicle (Figure 2C). Evidence of fewer interconnected cords and fragmented meshes was observed with 2,5-dihydroxy ketone analog (5) in both cell lines, whereas 3,4-dihydroxy ketone analog (4) only altered VM parameters in ES-2 cells (Figure 2C). Altogether, these data demonstrate that CAPE’s ketone analogs (4) and (5) can exert anti-VM activity in ovarian cancer cell models.

### 3.4 Dihydroxy ketone analogs (4) and (5) potently disrupt *in vitro* capillary-like structure formation and inhibit chemotactic cell migration in a concentration-dependent manner

**FIGURE 3:**
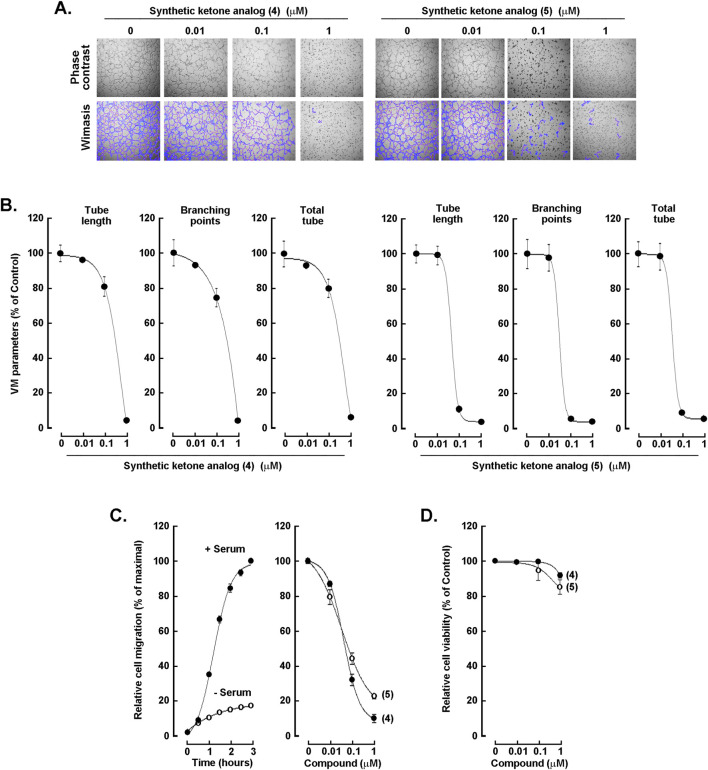
Synthetic ketone analogs (4) and (5) inhibit in vitro VM formation in a concentration-dependent manner. ES-2 cells were seeded on top of Matrigel in the presence of the indicated concentrations of analogs (4) or (5). **(A)** Representative phase-contrast images (upper panels) and corresponding Wimasis segmentation (lower panels) are shown. **(B)** Quantification of VM parameters, tube length, branching points, and total tubes expressed as percentage of the vehicle control. Data are the mean ± SD of 3 independent experiments, and asterisks (*) indicate statistically significant differences *versus* vehicle (DMSO) with *P* < 0.05 **(C)** Cell chemotaxis in response to serum (left panel) and in response to the indicated concentrations of analogs (4) or (5). **(D)** ES-2 cells were treated with the indicated concentrations of (4) or (5) for 24 h. Cell viability was evaluated for each concentration using the Trypan Blue exclusion assay and a TC20 Automated Cell Counter (Biorad). Data are representative of two independent experiments.

To further test whether ketone synthetic analogs (4) and (5) exerted dose-dependent anti-VM activity, capillary-like structure formation assay was performed and network architecture quantified in ES-2 cells. Representative phase-contrast images (Figure 3A, upper panels) show that increasing concentrations of either ketone analogs (4) or (5) progressively reduced network complexity, transitioning from well-connected meshes in vehicle-treated cells to sparse, fragmented structures at 0.1 µM and near-complete ablation at 1 µM as analyzed upon Wimasis (Figure 3A, lower panels). Quantitative analyses of tube length, branching points, and total tubes, expressed as percent of vehicle control and plotted as dose-response curves, reveal a steep decline by 0.1 µM across all metrics and minimal residual network formation at 1 μM, indicating robust, concentration-dependent inhibition of morphogenesis by both analogs (Figure 3B). Finally, ES-2 cell chemotaxis was assessed in the presence or absence of serum. Cell migration was found significantly increased in response to serum (Figure 3C, left panel, closed circles), whereas serum-induced chemotactic response was dose-dependently abrogated in the presence of either analog with IC_50_ values of 41.7 nM for (4) and 33.7 nM for (5) (Figure 3C, right panel). The observed low nanomolar potency strongly supports the drugability potential of these two analogs, as very high target affinity is typically associated with reduced dosing requirements and improved formulation flexibility, without affecting significantly cell viability (Figure 3D). Moreover, it offers a greater capacity to discriminate on-target effects from off-target cytotoxic liabilities ultimately favoring a wider therapeutic window.

### 3.5 Dihydroxy ketone analogs (4) and (5) broadly repress oncogenic, stemness, and inflammatory transcriptional programs

**FIGURE 4:**
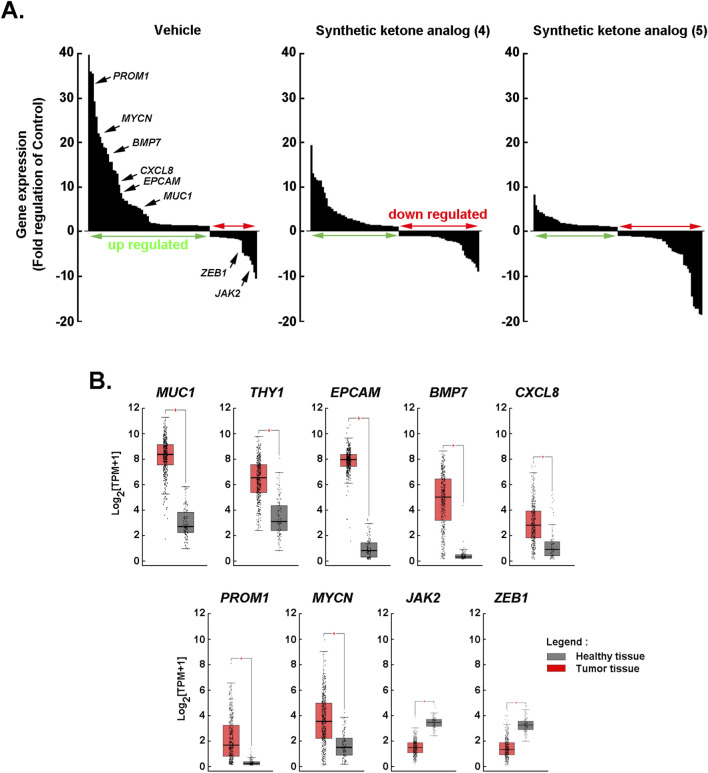
Global transcriptomic effects of synthetic ketone analogs (4) and (5). **(A)** Ranked waterfall plots of differential gene expression (log_2_ fold change relative to the vehicle), with green arrow indicating upregulated transcripts and red arrow indicating downregulated transcripts. Selected markers are annotated to illustrate shifts within the distribution. Both analogs (4) and (5) produce a broad modulation of the transcriptome compared with vehicle, with a net shift toward downregulation. **(B)** Box-and-whisker plots (log_2_ TPM) comparing expression in healthy tissue (grey) *versus* tumor tissue (red) for the indicated genes, illustrating disease-associated differences across epithelial/oncogenic, inflammatory, and EMT-related markers. Boxes denote the interquartile range with the median line; whiskers represent the data range.

To determine whether the CAPE-derived dihydroxy ketone synthetic analogs (4) and (5) modulate transcriptional networks linked to epithelial identity, stemness, EMT, and inflammation, we performed gene-expression profiling using a curated 89-gene panel under matched exposure conditions and compared drug-induced shifts with CSC-associated expression in ES-2-treated cells. Ranked “waterfall” distributions of log_2_ fold changes illustrate the global upregulated (green arrow) and downregulated (red arrow) transcriptomic response to ketone analogs (4) and (5) relative to the vehicle (Figure 4A). Annotated markers *BMP7, MUC1, EPCAM*, *PROM1*, *MYCN*, *JAK2*, *ZEB1*, and *CXCL8* highlight positions within the distribution with complete transcripts tested provided in the (Supplementary Figure S1). Both ketone analogs (4) and (5) produced a net shift toward transcriptional downregulation, with notable suppression across epithelial/stemness and inflammatory nodes, indicating broad pathway de-activation (Figure 4A). Box-and-whisker plots (log_2_ TPM) for several of the modulated markers were assessed in healthy tissue (Figure 4B, grey boxes) *versus* tumor tissue (Figure 4B, red boxes), showing higher tumor-associated expression for epithelial/stemness drivers (*EPCAM*, *PROM1*, *MYCN*, *THY1*) and inflammatory mediators (*CXCL8*) alongside decreased EMT regulators (*ZEB1*, *JAK2*). Taken together, the ranked response profiles and tissue comparisons suggest that 3,4- and 2,5-dihydroxy ketone (4) and (5) counteract transcriptional signatures enriched in tumors, consistent with a mechanism that dampens epithelial/stemness and inflammation while constraining EMT-linked plasticity all associated with VM (Supplementary Figure S1).

### 3.6 CAPE-derived ketone analogs (4) and (5) suppress stemness- and inflammation-associated transcripts across 2D monolayer and vasculogenic mimicry contexts

**FIGURE 5:**
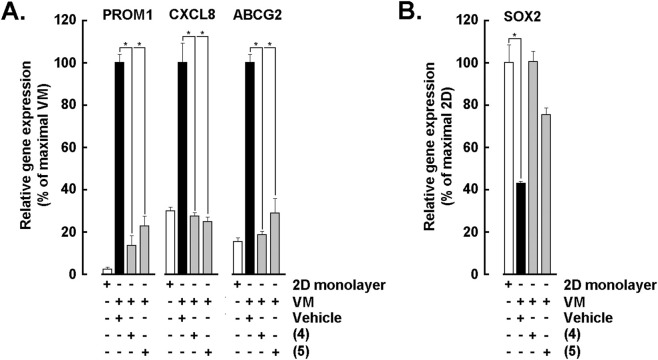
Synthetic ketone analogs (4) and (5) suppress stemness- and inflammation-associated gene expression across culture conditions. **(A)** Bar graphs of relative gene expression (%) for *PROM1*, *CXCL8*, and *ABCG2* measured in 2D monolayer cultures, vasculogenic mimicry (VM) networks, vehicle control, and cells treated with analogs (4) or (5). **(B)** Bar graph depicts *SOX2* expression under the same conditions, demonstrating decreased expression after exposure to analogs (4) and (5) relative to vehicle and untreated controls. Data are the mean ± SD of 3 independent experiments, and asterisks (*) indicate statistically significant differences *versus* vehicle (DMSO) with *P* < 0.05.

Building on the global modulation observed in the focused transcriptomic screen, we further wished to confirm by qPCR whether several of the individual markers linked to tumor cell plasticity (*PROM1/CD133*, *ABCG2*), stemness (*SOX2*), and inflammation (*CXCL8/IL-8*) are indeed directly modulated by the lead analogs. Bar plots of relative transcript abundance for *PROM1*, *CXCL8*, and *ABCG2* across 2D monolayer, VM, vehicle, and treatment with ketone analogs (4) and (5) demonstrates both analogs markedly reduced *PROM1* and *ABCG2* compared with vehicle and untreated baselines, while *CXCL8* was also consistently attenuated, indicating convergence on plasticity and inflammatory outputs (Figure 5A). In parallel, *SOX2* under the same conditions and upon treatment with (4) and (5) prevented lowering expression relative to vehicle, supporting a direct suppression of stemness circuitry by these chemotypes (Figure 5B). Together, these targeted readouts demonstrate that 3,4- and 2,5-dihydroxy ketone (4) and (5) reduce the transcript levels of key drivers of tumor cell self-renewal, drug-resistance, and pro-inflammatory signaling in VM settings. These interpretations are framed explicitly as hypotheses unless supported by future mechanistic experiments.

## 4 Discussion

Pharmacologically, CAPE (1) is relatively lipophilic and cell-permeable metabolite, which favors cellular uptake, but its systemic pharmacokinetics, metabolic stability, and long-term safety in humans remain incompletely defined; most evidence to date is preclinical, and translational development will eventually require improved formulations and rigorous toxicology studies. (Olgierd et al., 2021; Zhang et al., 2014). The selection of the four synthetic analogs (2–5) evaluated in this study was guided by a rational structure-activity relationship approach centered on CAPE (1), a plant-derived metabolite known for its diverse biological activities (Selka et al., 2022). CAPE (1) and its four closely related synthetic analogs (2–5) (Figure 1) were evaluated as a coherent series to probe how targeted structural variation modulates biological pathways linked to *in vitro* VM and cancer stemness. Extension of the alkyl chain in analog (2) (Figure 1A) enabled evaluation of the role of linker length and flexibility, whereas analog (3) (Figure 1B), incorporating a bioisosteric replacement of the ester function, provided insight into the functional relevance of this linkage and its contribution to the pharmacological profile. In parallel, 3,4- and 2,5-dihydroxy ketone analogs (4) and (5) (Figure 1A) were also evaluated to investigate the influence of dihydroxyl substitution patterns on the aromatic ring, thereby addressing how positional arrangement of the hydroxyl groups impacts the biological activity. This integrated approach allows a more nuanced understanding of structure-activity relationships within the CAPE (1) scaffold and highlights key features associated with differential modulation of VM and CSC phenotypes.

The two most biologically active synthetic analogs against VM were the ketone analogs (4) and (5) (Figure 2C) in which the ester oxygen was replaced by a methylene group. Notably, these two analogs differed only by the relative substitution of the two hydroxyl groups on the aromatic ring with analog (4) retaining the original 3,4-dihydroxylated (catechol) motif of CAPE (1) and analog (5) bearing a 2,5-dihydroxyl substitution pattern. This subtle but critical difference highlights the major influence of hydroxyl groups topology on this specific biological activity, underscoring that not only the presence but also the precise di-substitution arrangement of phenolic functionalities is a key determinant in VM inhibition (Figure 3).

Importantly, ketone analogs (4) and (5) exhibited identical molecular weights, TPSA, LogP values, and identical *n*-ROTB, *n*-HBA, and *n*-HBD profiles (Table 1). Taken together, the indistinguishable physicochemical properties of these two analogs indicate that their differential pharmacological effects are not driven by lipophilicity, polarity, or molecular flexibility, but rather by more subtle structural features. Despite these comparable physicochemical profiles, ketone analogs (4) and (5) exhibited markedly distinct biological behaviors toward CSC-associated VM pathways. The 2,5-dihydroxyled ketone (5) promoted a coordinated suppression of stemness and cellular plasticity programs, whereas the 3,4-dihydroxylated analog (4) only partially disrupted these networks. This divergence strongly suggests that the special orientation of the phenolic hydroxyl groups governs key molecular interactions involved in the regulation of our CSC line maintenance and VM, thereby overriding global physicochemical consideration.

Polyphenolic scaffolds have been described in the literature as potential pan-assay interference compounds (PAINS), particularly in biochemical assays where redox activity, aggregation, or non-specific protein interactions may generate artifactual signals (Magalhães et al., 2021; Bolz et al., 2021). These concerns are especially relevant for simple catechol-containing metabolites. However, several aspects of the present study argue against a purely non-specific interference mechanism. First, the biological evaluation relied on cell-based functional assays rather than isolated acellular enzyme systems, thereby reducing the likelihood of assay-specific redox artifacts. Second and more importantly, the synthetic ketone analogs (4) (3,4-dihydroxy) and (5) (2,5-dihydroxy) displayed divergent effects on CSC networks and VM despite exhibiting nearly identical molecular weights, lipophilicity, TPSA values, and hydrogen-bonding capacities. If the observed pharmacological activity were primarily driven by promiscuous redox reactivity or aggregation-based interference, comparable biological profiles would have been expected for these closely related regio-isomers. Instead, the differential transcriptional signature of stemness and plasticity phenotypes supports a structure-dependent pharmacological effect linked to hydroxyl positioning on the aromatic ring. While further mechanistic investigations will be required to fully elucidate molecular targets, the regioselective divergence observed here argues against a generalized PAINS-type behavior and supports the biological relevance of this scaffold in a cellular context.

3,4- and 2,5-Dihydroxy ketone analogs (4) and (5) further demonstrated a potentially compelling impact on the CSC phenotype in ovarian cancer by broadly repressing oncogenic, stemness, and inflammatory transcriptional programs that underpin tumor initiation, therapy resistance, and plasticity. Our gene expression profiling showed a net transcriptional downshift after exposure to the two analogs (4) and (5) (Figure 4A). This coordinated repression targeted the core molecular nodes that sustain CSC identity, self-renewal transcription factors, pro-survival signaling, and cytokine networks, thereby undermining the cellular programs that allow CSCs to survive cytotoxic stress, efflux drugs, and repopulate tumors after treatment. While the transcriptional results are consistent with CSC-associated pathways, impact on CSC biology needs to further be defined by functional behavior including self-renewal assays, limiting dilution tumorigenicity assays, CSC frequency measurements, or relapse modeling. Without functional validation, our data demonstrate transcriptional remodeling consistent with reduced stemness, but do not establish proven CSC depletion.

Mechanistically, the results indicate that 3,4- and 2,5-Dihydroxy ketone analogs (4) and (5) appear to act upstream of multiple CSC hallmarks rather than on a single downstream effector. By simultaneously dampening epithelial/stemness transcripts and inflammatory molecular signature, these two analogs are positioned to possibly reduce CSC frequency and functional capacity through several convergent effects including lowering expression of surface and transcriptional stemness markers that mark tumor-initiating cells, by decreasing cytokine-driven paracrine loops (IL-8/CXCL8) that may reinforce stemness and recruit supportive stroma; and by constraining EMT-linked plasticity that enables trans-differentiation into VM-competent phenotypes (Figure 5). The observed downregulation of *MYCN* and *PROM1* is particularly relevant because these factors are linked to proliferative drive and tumor-initiating potential as their suppression suggests a shift from a self-renewing, therapy-resistant state toward a more differentiated, therapy-sensitive phenotype. Finally, because Matrigel assays capture only a simplified, non-perfused microenvironment, inhibition of VM observed *in vitro* should be considered preliminary and may have limited direct implications for *in vivo* VM, which is shaped by tumor-stroma crosstalk, hemodynamics, and immune context. Thus, the anti-VM effects reported here would require confirmation in orthotopic and *in vivo* models.

The transcriptomic data reported here show broad transcriptional repression of CSC-related genes. Caution must be exerted as transcriptional changes alone do not demonstrate functional CSC depletion. Nevertheless, these transcriptional effects may predict several clinically meaningful outcomes. First, reducing CSC-associated programs could possibly lower the reservoir of cells capable of driving relapse. Second, suppression of inflammatory mediators and EMT regulators may enhance drug delivery and reduce microenvironmental protection, increasing chemosensitivity across the tumor mass rather than only within the CSC compartment. Third, as 3,4- and 2,5-Dihydroxy ketone analogs (4) and (5) target multiple nodes simultaneously, they may limit rapid adaptive resistance that commonly arises when single pathways are inhibited. Together, these properties support the development of 3,4- and 2,5-Dihydroxy ketone analogs (4) and (5) as possible adjuvants to standard chemotherapy or as combination partners with agents that target complementary vulnerabilities such as DNA-damage response inhibitors or ABC transporter modulators. Despite these promising signals, translational considerations and immediate clinical extrapolation must be tempered. The profiling data are transcriptomic and require functional validation, and reductions in *EPCAM* or *PROM1* mRNA must be correlated with decreases in *vivo* relapse rates in orthotopic or patient-derived xenograft models. Pharmacokinetic and pharmacodynamic relationships will be required to determine whether the transcriptional repression observed *in vitro* is achievable and durable *in vivo* at tolerable exposures. Off-target effects and potential impacts on normal progenitor populations will also require careful assessment, since broad suppression of stemness programs could affect tissue regeneration.

Looking forward and in conclusion, a pragmatic preclinical path would pair mechanistic assays with translational models. CSC frequency and functional assays after treatment with 3,4- and 2,5-Dihydroxy ketone analogs (4) and (5) tested in combinations with frontline chemotherapies to measure synergy and relapse prevention should be addressed to also evaluate effects on VM formation and tumor perfusion to link transcriptional changes to microenvironmental remodeling. If these steps confirm that transcriptional repression translates into functional CSC depletion and improved treatment durability, CAPE’s (1) synthetic analogs (4) and (5) would represent a rational strategy to dismantle the CSC reservoir and its protective niche in ovarian cancer, addressing a central mechanism of chemoresistance and relapse.

## Data Availability

The datasets used and/or analyzed during the current study are available from the corresponding authors upon reasonable request.

## Supplementary material

SUPPLEMENTARY FIGURE S1 Unsupervised clustering of the curated 89-gene panel reveals coordinated modules spanning epithelial identity, stemness/drug resistance, EMT, inflammation, and receptor signaling. To contextualize VM-induced transcriptional changes, we mapped the baseline organization of our marker set using hierarchical clustering of z-score–scaled expression values, anticipating that co-regulated genes would group into functional modules relevant to epithelial differentiation, plasticity, and signaling. The heatmap displays one row per gene with a left-hand dendrogram indicating cluster relationships; the color gradient (blue to red) denotes relative gene expression from low (−9) to high (34). Distinct branches capture epithelial markers (e.g., EPCAM, KRTs) and adhesion molecules (PECAM1, EPCAM), a stemness/drug-resistance cluster (PROM1/CD133, ABCG2, ALDH1A1, MYCN), EMT/mesenchymal regulators (ZEB1, SNAI1, TWIST1/2, VIM), inflammatory mediators (CXCL8/IL-8), and signaling nodes spanning Notch (NOTCH1/2, DLL1/4, JAG1), Hedgehog (WNT1, PTCH1, SMO), BMP (BMP7), receptor tyrosine kinases (FGFR2, AXL), and cytokine/JAK-STAT components (JAK2, STAT3). The clustering highlights coherent expression modules that underpin epithelial state, plasticity, and pathway activation, providing a structured framework to interpret subsequent drug-response analyses and to link targeted gene effects to emergent biological processes.

SUPPLEMENTARY TABLE S1 Biological processes identified by unsupervised clustering of the 89-gene panel. The table summarizes functional modules derived from z-score–standardized gene-expression values and hierarchical clustering. Six modules are described: a Suppressed Differentiation and Lineage-Committed Programs (low-expression block) featuring representative genes such as SOX2, JAK2, NOTCH2, B2M, ITGB1, ZEB1, ITGA6, MERTK, KLF4, LATS1, WWC1, and ALCAM and capturing repression of differentiation, loss of lineage programs, reduced immune signaling, and diminished mechanotransduction, with enrichment for Hippo, integrin, and interferon-associated cascades, consistent with a strongly inhibited transcriptional state; an Early Stem/Progenitor State and Epigenetic Maintenance (intermediate-low expression) set including ID1, BMI1, FOXP1, DNMT1, STAT3, RPLP0, and ENG that reflects epigenetic maintenance, transcriptional priming, and early progenitor identity, enriched for chromatin remodeling, STAT-mediated signaling, and regulatory maintenance programs; a Canonical Notch Signaling Activation (central signaling module) comprising JAG1, DLL4, TGFBR1, MYC, and KLF17 and denoting Notch receptor–ligand activation, cell–cell communication, and transcriptional modulation, with enrichment for Notch, TGFβ, and MYC-regulated pathways; an Adhesion/Extracellular Matrix/Mechanotransduction (intermediate-high expression) block with PECAM1, KIT, EPCAM, AXL, GSK3B, SMO, and TWIST1/2 highlighting cell adhesion, ECM interaction, mechanotransduction, and partial EMT readiness, enriched for Hedgehog, adhesion, and cytoskeletal regulation; a Migration/EMT/Plasticity (high-intermediate expression) group including CXCL8, SNAI1, ZEB2, THY1, PTCH1, and LIN28 A/B that supports mobility, cytokine-mediated remodeling, and early EMT-like plasticity, enriched for EMT, morphogen, and inflammatory cytokine pathways; and a High Stemness Signature: Hedgehog/BMP/Notch peak (highest expression block) defined by PROM1, FGFR2, NOS2, MS4A1, ABCB5, MYCN, DLL1, WNT1, ALDH1A1, BMP7, and ABCG2, indicating stem-cell maintenance, metabolic reprogramming, and peak Notch-Wnt-BMP activation with enrichment for Hedgehog, BMP, and Notch/Wnt signaling.
