# Supplementary material for: Targeting in vitro vasculogenic mimicry and associated stemness transcriptional signature in human ovarian cancer cell models: new emerging roles of caffeic acid phenethyl ester synthetic analogs
Source: Front Pharmacol. 2026 Mar 17;17:1787101. doi: 10.3389/fphar.2026.1787101 (PMC13036157; doi:10.3389/fphar.2026.1787101)
Supplement: Supplementary file 1 [file Supplementaryfile1.docx]

**Supplementary information**

**Targeting In Vitro Vasculogenic Mimicry and Associated Stemness Transcriptional Signatures in Human Ovarian Cancer Cell Models: New Emerging Roles of Caffeic Acid Phenethyl Ester Synthetic Analogs**

Mohamed Touaibia^1^, Anes Boudah^2^, Alain Zgheib^2^, Bogdan Alexandru Danalache^2^, and Borhane Annabi^2^

*^1^Department of Chemistry and Biochemistry, Université de Moncton, Moncton, NB, Canada, E1A 3E9, and ^2^Laboratoire d’Oncologie Moléculaire, Département de Chimie, Université du Québec à Montréal, Montreal, QC, Canada, H3C 3P8*

**Content**

1. General synthetic experimental procedures …………………………………………S2

2. Synthesis of ketone analog (**4**) …………………………...……………………….…S2

3. References ………...…………………………………...………….……..………..…S3

**1. General synthetic experimental procedures**

All reagents and chemicals were purchased from Millipore Sigma and used without further purification unless otherwise noted. Purification procedure was carried out with reagent-grade solvents in air. Thin layer chromatography (TLC) analysis was conducted using silica gel-coated aluminum sheets (SiliaPlate TLC, Silicycle®) with detection by UV light (254 nm, UVS-11, Mineralight® shortwave UV lamp). Purification was carried out by flash chromatography (Isco, Inc. CombiFlash™ Sg100c). 1H and 13C nuclear magnetic resonance (NMR) spectra were recorded at room temperature using Bruker AV-III-400 spectrometer. Chemical shifts (δ values) were reported in parts per million and were referenced to the deuterated residual solvent peak. NMR data were reported as δ value (where s = singlet, d = doublet, t = triplet, q = quartet, and quin = quintuplet, integration, J value (Hz)). The value of the coupling constant (J = 15-16 Hz) confirms the trans stereochemistry of α,β-unsaturation. High-resolution mass spectrometry (HRMS) measurements were performed on an Agilent 6200 high-resolution time-of-flight mass spectrometer equipped with a Dual ESI ion source.

**2. Synthesis of ketone analog** (**4**)

3,4-dihydroxybenzaldehyde (250 mg, 1.81 mmol), 5-phenylpentan-2-one (325 mg, 1.99 mmol, 1.1 eq.), and five drops of acetic acid mixed with 10 mL of tetrahydrofuran, and pyrolidine (100 µl) were used as starting reagents. The reaction was carried out following the same procedure as that described for the optimized synthesis of ketone analog (**5**) (Giordano et al., 2025; Touaibia et al., 2022). After extraction, and purification by flash chromatography with hexane/ethyl acetate (7:3, v/v), analog (**4**) was obtained as a yellow solid (mp: 136-138 ^o^C, 419 mg; Yield: 82%. ^1^H NMR (400 MHz, DMSO) δ 9.59 (s, 1H, OH), 9.14 (s, 1H, OH), 7.42 (d, *J* = 16.2 Hz, 1H, =CH-Ar), 7.33-7.25 (m, 2H, H_ar_), 7.23-7.15 (m, 3H, H_ar_), 7.07 (d, *J* = 2.1 Hz, 1H, H_ar_), 7.01 (dd, *J* = 8.2, 2.1 Hz, 1H, H_ar_), 6.77 (d, *J* = 8.2 Hz, 1H, H_ar_), 6.56 (d, *J* = 16.2 Hz, 1H, =CH-CO), 2.69-2.62 (m, 2H, CH_2_-CO), 2.59 (t, *J* = 7.7 Hz, 2H, CH_2_-Ar), 1.85 (p, *J* = 7.3 Hz, 2H, CH_2_-CH_2_-CH_2_);^13^C NMR (101 MHz, DMSO) δ 199.88, 148.89, 146.07, 143.19, 142.30, 128.77, 126.30, 126.25, 123.52, 122.06, 116.26, 115.30, 35.05, 26.30; HRMS cal. for C_18_H_18_O_3_ + (H^+^): 283.1334. Obtained 238.1331.

**3. References**

Giordano C, Kendler J, Sexl M, Kollman S, Varenicja M, Szabó B, et al. (2025) Anti-cancer potential of a new derivative of caffeic acid phenethyl ester targeting the centrosome. *Redox Biol*. 81, 103582. doi: 10.1016/j.redox.2025.103582

Touaibia M, Faye DC, Doiron JA, Chiasson AI, Blanchard S, Roy PP, et al. (2022) Structure–activity relationship studies of new sinapic acid phenethyl ester analogues targeting the biosynthesis of 5-lipoxygenase products: the role of phenolic moiety, ester function, and bioisosterism. *J. Nat. Prod*. 85, 225-236. doi: 10.1021/acs.jnatprod.1c00982
