# Supplementary figures and images for: Targeting in vitro vasculogenic mimicry and associated stemness transcriptional signature in human ovarian cancer cell models: new emerging roles of caffeic acid phenethyl ester synthetic analogs
Source: Front Pharmacol. 2026 Mar 17;17:1787101. doi: 10.3389/fphar.2026.1787101 (PMC13036157; doi:10.3389/fphar.2026.1787101)

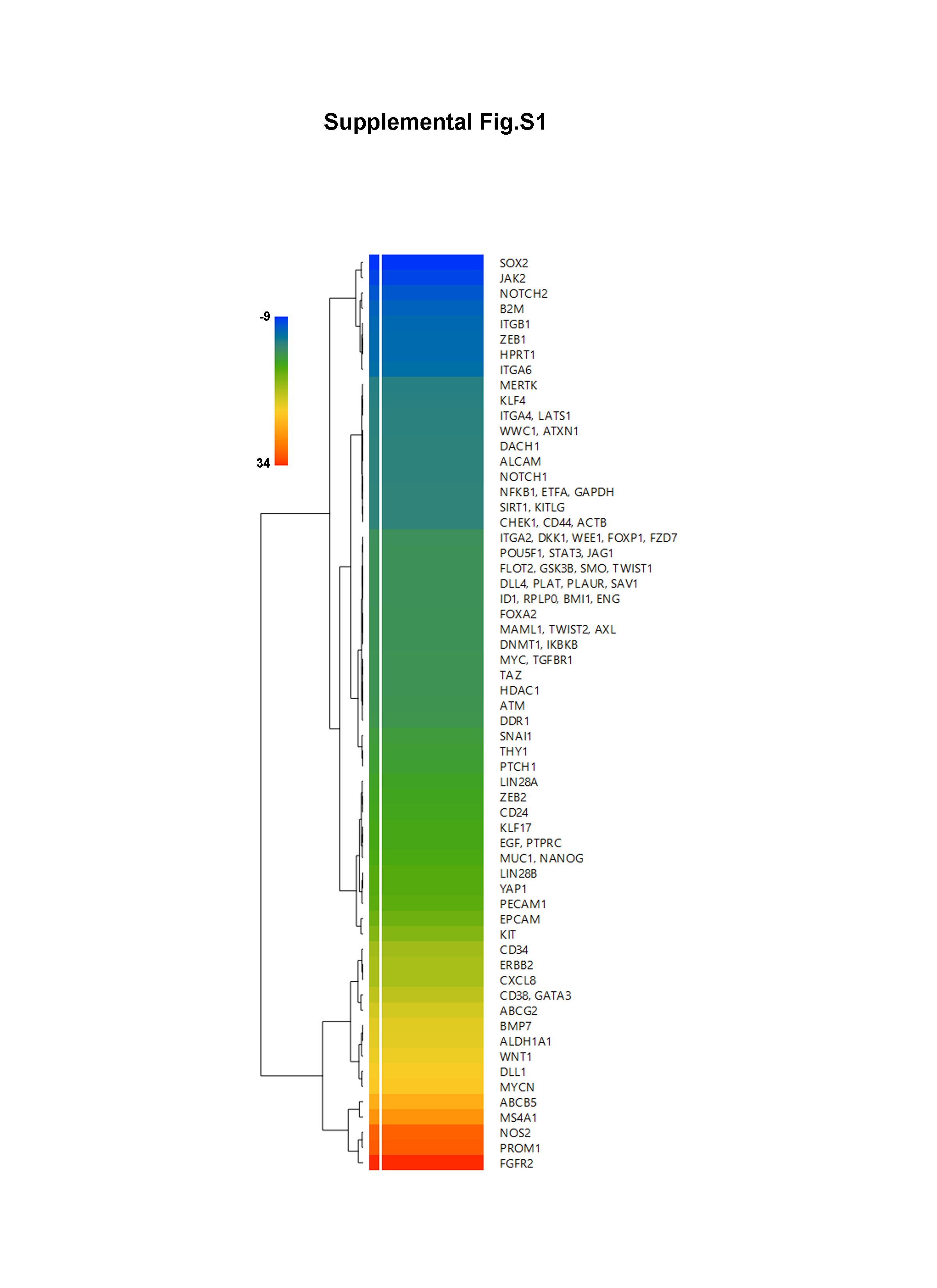

Supplement: Supplementary file 2 [file Image1.tif]
